# Risk Factors Associated with False Positive HIV Test Results in a Low-Risk Urban Obstetric Population

**DOI:** 10.1155/2012/841979

**Published:** 2011-08-11

**Authors:** Tamara T. Chao, Jeanne S. Sheffield, George D. Wendel, M. Qasim Ansari, Donald D. McIntire, Scott W. Roberts

**Affiliations:** ^1^Department of Obstetrics and Gynecology, The University of Texas Southwestern Medical Center, Dallas, TX 75390-9032, USA; ^2^Department of Pathology, The University of Texas Southwestern Medical Center, Dallas, TX 75390-9032, USA

## Abstract

*Objective*. To examine risk factors for false positive HIV enzyme immunoassay (EIA) testing at delivery. *Study Design*. A review of pregnant women who delivered at Parkland Hospital between 2005 and 2008 was performed. Patients routinely received serum HIV EIA testing at delivery, with positive results confirmed through immunofluorescent testing. Demographics, HIV, hepatitis B surface antigen (HBsAg), and rapid plasma reagin (RPR) results were obtained. Statistical analyses included Pearson's chi-square and Student's *t*-test. *Results*. Of 47,794 patients, 47,391 (99%) tested negative, 145 (0.3%) falsely positive, 172 (0.4%) positive, and 86 (0.2%) equivocal or missing HIV results. The positive predictive value of EIA was 54.3%. Patients with false positive results were more likely nulliparous (43% versus 31%, *P* < 0.001) and younger (23.9 ± 5.7 versus 26.2 ± 5.9 years, *P* < 0.001). HIV positive patients were older than false positive patients and more likely positive for HBsAg and RPR. *Conclusion*. False positive HIV testing at delivery using EIA is associated with young maternal age and nulliparity in this population.

## 1. Introduction

The Centers for Disease Control and Prevention (CDC) and United States Preventive Services Task Force (USPSTF) recommend universal HIV screening for all pregnant women entering prenatal care [1, 2]. This screening enables HIV-infected women and their infants to benefit from appropriate and timely interventions such as antiretroviral medications. When the recommended antiretroviral and obstetric interventions are used, a woman who knows of her HIV infection early in pregnancy now has a less than 2% chance of delivering an HIV-infected infant. Without intervention, this risk is approximately 25% in the United States [3–6]. 

Testing for HIV began in 1985 with the introduction of the enzyme immunoassay (EIA). In order to account for false positive results using screening tests in a low-prevalence population, confirmatory testing has been implemented using a Western blot or immunofluorescence assay. In a low-prevalence population, the false positive rate using the EIA is increased compared to a high-prevalence population, and the positive predictive value of any test will always depend on the prevalence of the condition in the population tested. In testing performed by the CDC, the EIA positive predictive value ranges from 71 to 83% in populations with HIV prevalence from 0.5 to 1% [7].

Pregnancy has been observed to be associated with false positive HIV testing. Some investigators believe that the presence of alloantibodies account for the increased false positive rate associated with pregnancy, transfusions, transplantation, and autoimmune diseases [8]. However, risk factors specific to pregnancy that account for this are poorly understood. Conversely, a recent large retrospective study found that the false positive HIV EIA rate was lower in pregnant women compared to nonpregnant individuals (0.14% versus 0.21%) [9]. Our objective was to determine if any maternal characteristics correlated with false positive HIV EIA testing at delivery.

## 2. Materials and Methods

This was a review of all women who delivered at Parkland Memorial Hospital in Dallas, Tex, from October 1, 2005 through September 30, 2008. All women routinely received serum HIV testing at their initial prenatal visit and at time of presentation to labor and delivery for delivery via the opt-out approach with the Abbott Commander HIV AB HIV-1/HIV-2 (rDNA) EIA (Abbott Laboratories, Abbott Park, Ill). HIV test results performed at the time of delivery were analyzed in this study. A woman was considered HIV negative if EIA testing was negative. Positive test results were confirmed with the fluorognost immunofluorescent assay (IFA) HIV-1 (Sanochemia Corp, Stamford, Conn, USA). Women were considered to be falsely positive if EIA results were positive and the IFA was negative. Women delivered in this time period were identified through the obstetric operations database and linked to the pathology database for HIV, hepatitis B surface antigen (HBsAg), and rapid plasma reagin (RPR) results. Maternal characteristics, including race, parity, age, singleton versus multiple gestation, and a diagnoses of diabetes or hypertension were obtained using the obstetrics operations database. Laboratory results drawn 28 days prior to delivery through seven days after delivery were included. 

The study was approved by the Institutional Review Board at the University of Texas Southwestern Medical Center. Categorical data were reported as frequencies, and statistical significance was determined using  *χ* ^2^ analysis. Two-group comparisons were made using Student's *t*-test. *P* values less than 0.05 were considered statistically significant. Statistical analyses were performed using SAS, Version 9.2 (SAS Institute, Cary, NC). 

## 3. Results

A total of 47,794 women were identified who delivered during the study time frame. Demographic and obstetrical characteristics of the patient population are shown in Table 1. Compared to HIV negative patients, false positive patients were more likely to be nulliparous (43% versus 31%, *P* < 0.001) and younger (mean age 23.9 ± 5.7 versus 26.2 ± 5.9, *P* < 0.001). HIV positive patients were significantly older than false positive patients (27.4 versus 23.9, *P* < 0.001) and HIV negative patients (27.4 versus 26.2, *P* = 0.012).

Of the 47,794 women, 47,391 (99%) were HIV negative, 145 (0.3%) had a false positive test, 172 (0.4%) were true positives, and 86 (0.2%) tested equivocal or were missing HIV results. The specificity of the HIV EIA test was 99.7% with a positive predictive value of 54.3%. The sensitivity of the EIA test was presumed to be near 100%, as the false negative rate using the EIA has been previously demonstrated to be negligible in studies performed by the manufacturer [10]. 

Table 2 shows the prevalence of diabetes, hypertension, hepatitis B, and results of syphilis testing in the study population. HIV positive women were more likely to test positive for hepatitis B surface antigen (1% versus 0%, *P* = 0.002) and RPR (2% versus 0%, *P* = 0.02). There was no significant difference in the prevalence of diabetes or hypertension between the three groups. There was also no significant difference in the rate of HBsAg and RPR positivity between the HIV negative and false positive groups.

When evaluating the interaction between nulliparity and age, there is a significant correlation between the two variables; that is, parous patients are likely to be older and, therefore, nulliparity and age are not independent predictors of HIV false positivity. However, when the HIV testing groups (positive, false positive, negative) are stratified by parity, the comparison of age across the three groups remains significant only in nulliparous women (Table 3, *P* = 0.0003). The interaction between parity and age means that the difference in age between nulliparous and parous women is different depending on the HIV status. For example, in the HIV positive group the nulliparous and parous women are closer in age than in the false positive group.

## 4. Comment

This was the first population-based study to evaluate risk factors for HIV false positivity in pregnant women presenting for delivery at a large urban institution. We found that younger and nulliparous women were more likely to have false positive testing using the HIV EIA. The observed positive predictive value (PPV) of 54.3% was lower than the previously reported by the CDC (PPV 71–83%) in a nonpregnant population [7], suggesting that pregnancy may be associated with a higher rate of false positivity. The low positive predictive value of the HIV EIA in our study may have been impacted by the relatively low HIV prevalence (0.4%) in our population. This information could be useful for counseling women who test positive for HIV at delivery and emphasizes the limitations of EIA testing if used as a rapid test to determine the need for intrapartum and neonatal antiretroviral prophylaxis.

The false positive rates of currently available rapid HIV tests have been reported to be much lower than has been found with EIA testing in this study. Tung et al. evaluated the HIV false positive rate in over 900 pregnant women, most of whom were Hispanic, using both the EIA and a rapid point-of-care (POCT) test, OraQuick [11]. They found that while there were no false positive tests with the OraQuick, there were seven false positives using the EIA (PPV 100% versus 35.7%). In the Mother-Infant Rapid Intervention at Delivery (MIRIAD) study, Jamieson et al. found that the PPV of the OraQuick test was 90% while the PPV of the EIA was 74% [12, 13].

Current recommendations by the CDC and the American College of Obstetricians and Gynecologists include rapid HIV screening for women presenting to labor and delivery with undocumented HIV status and for repeat HIV testing in the third trimester for women at high risk or who live in areas with high HIV prevalence [7, 14, 15]. A woman testing preliminarily positive for HIV in labor should be counseled that she may have HIV infection and that her neonate may be exposed, and immediate antiretroviral prophylaxis should be recommended without waiting for confirmatory test results. The results of our study may aid in counseling women if they test preliminarily positive for HIV using the EIA, while awaiting confirmatory testing results. It remains to be determined whether the same risk factors for false positive HIV EIA testing apply to the POCT tests used in a real life setting.

Our study found that the positive predictive value of EIA testing was only 54.3% and that younger nulliparous women were more likely to test falsely positive. The reasons for these findings are not entirely clear. Importantly, our data may represent the real world performance of the EIA testing in a large obstetric population-based setting. Investigators also have noted a significant relationship between influenza vaccination and false positive screening for HIV antibodies [16–18]. A potential reason for this cross reactivity is that there are similarities in homology between the transmembrane domains of the influenza envelope protein hemagglutinin and the HIV-1 envelope proteins [19]. In our population, there were no significant differences in age or parity and influenza vaccination acceptance rates (unpublished data).

There were several limitations to our study. Women in our study were from a single institution, with a predominantly Hispanic background, and therefore the results may not be applicable to all populations. Reasons for false positive HIV serology may vary depending on the geographical region. While we did find a significant association between young age, nulliparity, and HIV false positive testing, our study does not have the capability to identify a causal relationship or explain why this association may exist. In addition, our study cannot address if these same risk factors for false positive testing apply to all other HIV tests. Further studies are needed to confirm our findings, elucidate the biological mechanisms for increased HIV EIA false positivity in young, nulliparous women, and compare this conventional testing method with contemporary rapid screening assays, including POCT.

## Figures and Tables

**Table 1:** Demographic and obstetric characteristics of HIV positive, false positive, and negative women.

Characteristic	Positive *N* = 172	False positive *N* = 145	Negative *N* = 47391	*P*		
				Positive versus false positive	Positive versus negative	False positive versus negative
Age: mean ± std	27.4 ± 6.2	23.9 ± 5.7	26.2 ± 5.9	<0.001	0.012	<0.001

Age				<0.001	0.021	0.002
<18	2 (1)	16 (11)	2494 (5)			
18–35	152 (88)	124 (86)	41412 (87)			
>35	18 (10)	5 (3)	3485 (7)			

Race				<0.001	<0.001	0.102
Black	116 (67)	19 (13)	4422 (9)			
White	16 (9)	8 (6)	1955 (4)			
Hispanic	38 (22)	118 (81)	39975 (84)			
Other	2 (1)	0 (0)	1039 (2)			

Nulliparous	45 (26)	63 (43)	14488 (31)	0.001	0.210	<0.001

Parity				0.002	0.009	0.004
0	45 (26)	63 (43)	14488 (31)			
1	53 (31)	47 (32)	14732 (31)			
2	35 (20)	21 (14)	10690 (23)			
3	19 (11)	9 (6)	4893 (10)			
>3	20 (12)	5 (3)	2588 (5)			

Multiple gestation	1 (1)	0 (0)	531 (1)	0.358	0.502	0.200

Data expressed as *N* (%) or mean ± standard deviation.

**Table 2:** Comorbidities, hepatitis B, and RPR results in HIV positive, false positive, and negative women.

Characteristic	Positive *N* = 172	False positive *N* = 145	Negative *N* = 47391	*P*		
				Positive versus false positive	Positive versus negative	False positive versus negative
Diabetes	13 (8)	8 (6)	3015 (6)	0.467	0.521	0.677
Hypertension	20 (12)	16 (11)	4164 (9)	0.868	0.189	0.340
HBsAg				0.002	<0.001	0.580
No result	12 (7)	0 (0)	224 (0.5)			
Negative	158 (92)	145 (100)	47038 (99)			
Positive	2 (1)	0 (0)	129 (0.3)			
RPR				0.020	<0.001	0.618
No result	5 (3)	0 (0)	31 (0.07)			
Nonreactive	163 (95)	145 (100)	47078 (99)			
Reactive	4 (2)	0 (0)	282 (0.6)			

Data expressed as *N* (%).

**Table 3:** HIV testing groups stratified by age and parity.

Nulliparous			
HIV result	≤19 years *N* = 4925	>19 years *N* = 9671	*P*
Positive	10 (0.20)	35 (0.36)	
False positive	35 (0.71)	28 (0.29)	0.0003
Negative	4880 (99.1)	9608 (99.4)	

Parous			

HIV result	≤19 years *N* = 1568	>19 years *N* = 31544	*P*
Positive	7 (0.45)	120 (0.38)	
False positive	6 (0.38)	76 (0.24)	0.5
Negative	1555 (99.2)	31348 (99.4)	

Data expressed as *N* (%).
